# Survival Outcomes for US and Canadian Patients Diagnosed with Hodgkin Lymphoma before and after Brentuximab Vedotin Approval for Relapsed/Refractory Disease: A Retrospective Cohort Study

**DOI:** 10.3390/curroncol31070287

**Published:** 2024-07-04

**Authors:** Gwynivere A. Davies, John E. Orav, Kristen D. Brantley

**Affiliations:** 1 Hamilton Health Sciences, Juravinski Cancer Centre, Hamilton, ON L8V 5C2, Canada; 2 Department of Oncology, McMaster University, Hamilton, ON L8S 4L8, Canada; 3 Department of Biostatistics, Harvard T.H. Chan School of Public Health, Boston, MA 02115, USA; eorav@bwh.harvard.edu; 4 Department of Epidemiology, Harvard T.H. Chan School of Public Health, Boston, MA 02115, USA; kbrantley@g.harvard.edu

**Keywords:** Hodgkin disease, outcomes research, insurance

## Abstract

Cost-effectiveness analyses are required for therapies within Canada’s universal healthcare system, leading to delays relative to U.S. healthcare. Patients with Hodgkin lymphoma (HL) generally have an excellent prognosis, but those who relapse after or are ineligible for transplant benefit from novel therapies, including brentuximab vedotin (BV). BV was FDA-approved in 2011 but not Canadian-funded until 2014. To assess the impact of access delays, we compared changes in survival for U.S. (by insurer) and Canadian patients in periods pre/post-U.S. approval. Patients were 16–64 years, diagnosed with HL in 2007–2010 (Period 1) and 2011–2014 (Period 2) from the U.S. SEER and Canadian Cancer Registries. Approval date (surrogate) was utilized as therapy was unavailable in registries. Kaplan-Meier survival curves and adjusted Cox regression models compared survival between periods by insurance category. Among 12,003 U.S. and 4210 Canadian patients, survival was better in U.S. patients (adjusted hazard ratio (aHR) 0.87 (95%CI 0.77–0.98)) between periods; improvement in Canadian patients (aHR 0.84 (95%CI 0.69–1.03) was similar but non-significant. Comparisons between insurers showed survival was significantly worse for U.S. uninsured and Medicaid vs. U.S. privately insured and Canadian patients. Given the increasingly complex nature of oncologic funding, this merits further investigation to ensure equity in access to therapy developments.

## 1. Introduction

Insurance status impacts treatment access and survival for cancer patients [1,2,3,4,5,6,7,8]. This can be due to factors including increased comorbidities and unhealthy behaviors, delayed or late-stage diagnosis and treatment, inability to navigate or mistrust of health care systems, lack of transportation or time off work, and lower-quality treatment by providers serving uninsured patients or those receiving Medicaid. In a 1999–2004 study of New Jersey patients diagnosed with the seven most common forms of cancer, uninsured patients or those receiving Medicaid had a 21–198% higher risk of death at 5 years for almost all types of cancer compared to privately insured patients [9]. Additionally, while privately insured patients with breast, colorectal, or lung cancer and non-Hodgkin lymphoma (NHL) experienced improved survival over time, there was no improvement for uninsured patients. A National Cancer Data Base study of 45,777 patients diagnosed with Hodgkin lymphoma (HL) between 1998–2011 similarly showed that patients with unfavorable insurance status (i.e., those on Medicaid or lacking insurance, often due to limited income) not only presented at a more advanced stage, had higher comorbidity scores, and were more likely to have constitutional symptoms (fevers, sweats, weight loss) but also were less likely to receive radiotherapy or start chemotherapy promptly and were less commonly treated at an academic or research center [1]. These differences translated to a 5 year overall survival (OS) of 54% vs. 87% for unfavorably vs. favorably insured, which retained significance after adjusting for covariates. Newer oncologic therapies including immunotherapy can be prohibitively expensive and thus concern arises that survival inequities will be accentuated. This can occur even in universal healthcare systems such as Canada, where drug approval and coverage are delayed, often by 1.5–2 years minimum, due to multi-level (national and provincial) governmental review, prolonged cost-effectiveness analyses and negotiations, and subsequent formulary deliberations [10,11].

There are 8570 new cases of HL and 910 HL deaths yearly in the U.S. [12]; within Canada, there were an estimated 1100 new cases and 110 deaths in 2023 [13]. While the five-year median OS (mOS) for adults is 88%, survival after relapse, especially post autologous stem cell transplant (ASCT), remained poor (mOS 10.5 to 27.6 months) with limited salvage options prior to 2011 [14]. Brentuximab vedotin (BV), an antibody drug conjugate, was the first new FDA-approved (August 2011) treatment for HL since 1977. Monotherapy demonstrated a response rate of 75%, with a 5 year OS of 41% (mOS = 40.5 months, 95% CI = 28.7–61.9 months) in a phase 2 study of patients relapsing post ASCT [14]. While this represents a significant improvement over the previous standard of care, significant equity concerns exist due to cost [15]. BV costs CAD 14,520 per treatment (for a 70 kg adult; weight-based dosing) within Canada and is provided up to a maximum of 16 cycles (CAD 232,320, 2018 dollars) [15]. This cost is even higher within the U.S. Given the challenge of funding these medications within the public system, significant stakeholder engagement, and cost efficacy analyses occurred prior to approval of this medication within Canada, creating a 3 year delay following FDA approval before this was eventually approved and funded for post-transplant relapse in Canada. Transplant-ineligible patients with similar poor survival had to wait several more years longer for funding, unlike in the U.S.

We therefore compared OS for U.S. and Canadian patients diagnosed with HL in time periods pre- and post-FDA approval of BV for post-transplant relapse, hypothesizing that (1) survival differences within the U.S. according to insurance status would be present and widen after approval and (2) a survival gap would emerge between privately insured U.S. vs. Canadian patients due to earlier access.

## 2. Materials and Methods

A retrospective cohort study was performed including two separate cohorts with data from (1) the U.S. SEER database and (2) the Canadian Cancer Registry. The SEER database is a national surveillance program that collects and publishes cancer and survival data, covering 48% of the U.S. population [16], with yearly updates of vital status. Baseline demographics—including age at diagnosis, sex, race, Hispanic status, marital status, and insurance status—were collected, as were lymphoma-specific variables, including date of diagnosis, subtype, stage, and outcome data including vital status (from the National Death Index). Since 2007 SEER has categorized individuals’ insurance status as: uninsured, any Medicaid, insured, insured “no specifics”, or insurance status unknown. The 2019 dataset was utilized.

The Canadian Cancer Registry (CCR) covers > 95% of the Canadian cancer population and is maintained by Statistics Canada with rigorous quality assurance auditing. Baseline demographics collected include age at diagnosis and sex (other variables from SEER dataset not available in CCR dataset), along with date of diagnosis, subtype, and outcome data. Vital status has most recently been updated to December 2017. Staging information is not available in approximately 75% of Canadian patients, limiting sensitivity analysis in this cohort. Statistics Canada requires rounding for distribution of data.

U.S. and Canadian patients aged 16–64 years diagnosed with classical HL in 2007–2010 (Period 1) or 2011–2014 (Period 2) were included in the analytic cohort. As neither dataset captures chemotherapy, a surrogate date for access (FDA approval of BV) was used to delineate the time periods of exposure (before and after presumed BV access in the U.S.; Canadian patients could not universally access BV at relapse until after 2014). Additionally, 72% of relapses occur within the first two years from diagnosis. Thus, these should occur mainly within the described time periods and follow-up [17]. Exclusion criteria included missing histology, diagnosis on autopsy/death certificate only, follow-up of zero/could be zero months, or unknown insurance status.

Baseline characteristics are described by medians (interquartile ranges), means (standard deviations), and proportions and compared by chi-square (dichotomous variables) and *t*-tests (continuous variables). The primary outcome was overall survival, measured from date of diagnosis until death or end of follow-up. Differences in survival by insurance type and by time period of diagnosis were evaluated using Kaplan–Meier analysis. For the analysis, U.S. insurance status was categorized as insured (includes both categories of insured within SEER), uninsured, or Medicaid. All patients within CCR were considered universally insured. Cox proportional hazards models were used to calculate adjusted hazard ratios comparing insurance types.

Survival analysis by period was initially performed within each country dataset to allow for maximal adjustment by covariates (age, gender, race, ethnicity, marital status, insurance status, stage, lymphoma subtype, within SEER; age, gender, lymphoma subtype within CCR), then U.S and Canadian data were merged using common variables (age, gender, lymphoma subtype). Proportional hazards were tested globally, and if violated, individual covariates were assessed for inclusion as time varying covariates (TVCs). HRs are presented with 95% confidence intervals (CI), and significance determined as *p* < 0.05. Sensitivity analysis was performed, restricted to advanced-stage (stage III/IV) patients, who have a higher risk for relapse (U.S. patients only); 60-month OS was assessed as a secondary outcome using the Nelson–Aalen estimate cumulative hazard function values to compare the direction and degree of change in survival between time periods and to determine whether gaps in survival between insurance types increased over time.

Missing data for covariates are reported in tables, but not imputed. All analyses were performed using Stata IC version 15.1 (U.S. data), or Stata BE version 18 (Canadian and merged data) as Canadian analyses were performed onsite at a Research Data Centre, as required by Canadian federal law.

Research ethics board approval was not required to proceed; however, both registries required data usage applications and agreements, with an extensive security review by Statistics Canada.

## 3. Results

### 3.1. Baseline Characteristics at HL Diagnosis

In total, 12,003 U.S. and 4210 Canadian patients were included (demographics detailed in Table 1). Specifically within the SEER database, we identified 6204 and 5799 U.S. patients in Time Periods 1 and 2, respectively. Mean age was 35.8 (standard deviation (SD) 13.5) years, 46.1% of patients were female, and approximately 80% were White. Insurance coverage and stage at diagnosis were similar between time periods. Follow-up was 83.7 (SD 28.8) months for Period 1 and 42.7 (SD 17.4) months in Period 2.

Within the Canadian Cancer Registry, 2370 and 1840 patients were identified during Periods 1 and 2, respectively. The number of patients was substantially lower in Period 2, as the province of Quebec stopped contributing data in 2011. Mean age was 35.8 (SD 13.7) years and 45.8% of patients were female in Period 1; patients were slightly younger with fewer females in Time Period 2, potentially due to data non-contribution and provincial demographics. Mean follow-up time was 99.2 (SD 29.1) months in Period 1 and 57.3 (SD 17.4) months in Period 2.

### 3.2. Outcomes from the Time of HL Diagnosis

Within the U.S. cohort, age, gender, race, ethnicity (Hispanic vs. non-Hispanic), marital status, stage, lymphoma subtype, and insurance status were all found to impact survival. Within the Canadian dataset, age, gender, and lymphoma subtype impacted survival, though location of diagnosis (province or provincial region/territory) did not. Stage was also found to be associated with survival; however, due to the large proportion of missingness for stage, this was not included in the final model. Therefore, when combining country datasets to compare universally covered (Canadian) patients with U.S. patients, age, gender, and lymphoma subtype were the covariates included.

**Figure 1:**
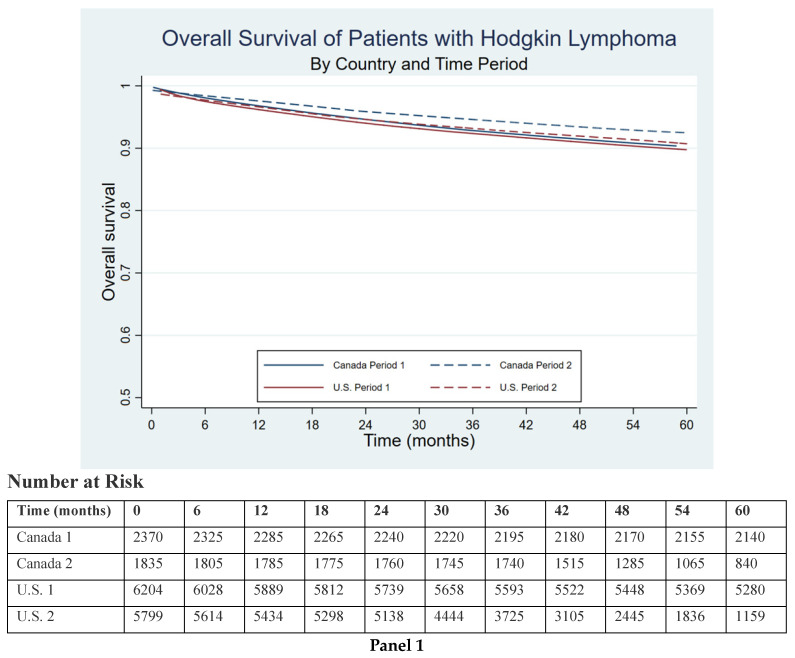

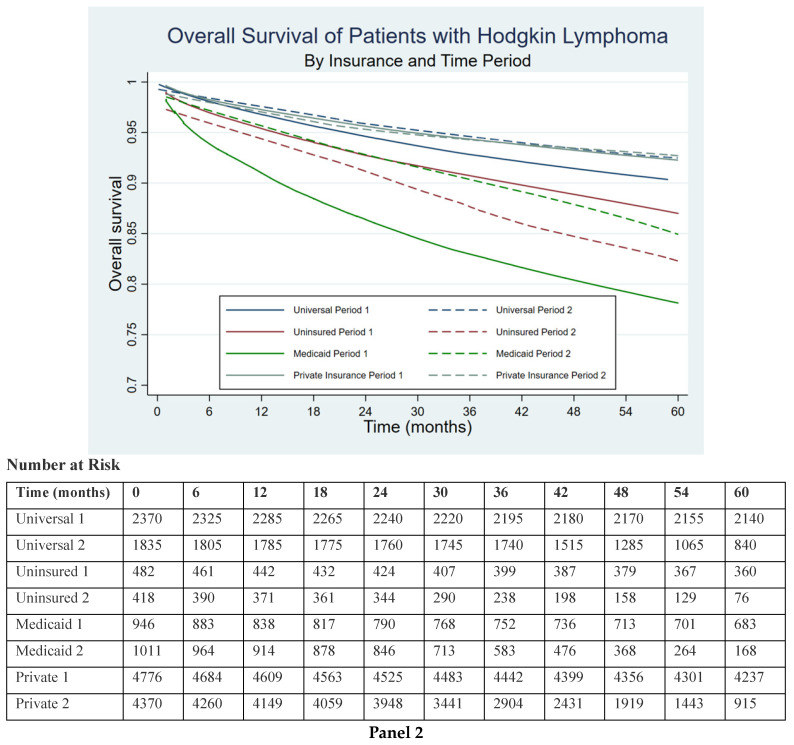
(**Panel 1**) Overall survival of U.S. and Canadian patients with Hodgkin lymphoma diagnosed during Time Period 1 (2007–2010) and Time Period 2 (2011–2014) during the first 60 months of follow-up from date of diagnosis. (**Panel 2**) Overall survival of U.S. and Canadian patients with Hodgkin lymphoma diagnosed during Time Period 1 (2007–2010) and Time Period 2 (2011–2014) according to insurance status during the first 60 months of follow-up from diagnosis.

U.S. HL patients diagnosed in Period 2 experienced better survival than those diagnosed in Period 1 (crude HR (95% CI) = 0.90 (0.80–1.02), *p* = 0.090; adjusted HR = 0.87 (0.77–0.98), *p* = 0.025), whereas the improvement in survival seen for Canadian patients became statistically insignificant after adjusting for age, gender, and lymphoma subtype (crude HR = 0.79 (0.64–0.97), *p* = 0.025, adjusted HR = 0.84 (0.69–1.03), *p* = 0.10) (Table 2; Figure 1). Maximally adjusting U.S. patients for all known covariates results in further survival improvements between periods (adjusted HR = 0.80 (0.71–0.91), *p* < 0.001).

In examining U.S. patients stratified by insurance status, divergence in survival over time was seen with stable OS for privately insured (adjusted HR = 0.93 (0.79–1.10), *p* = 0.39), significantly higher OS in Period 2 for patients with Medicaid (adjusted HR = 0.50 (0.39–0.64), *p* < 0.001), and non-significantly but suggestively lower survival in Period 2 for uninsured patients (adjusted HR 1.30 (0.88–1.91), *p* = 0.187). Values were similar when restricted to advanced-stage (stage III and IV) U.S. patients only (Supplementary Materials).

A significantly increased risk of death was seen for U.S. patients compared to Canadian patients when combining data from both time periods (crude HR = 1.14 (1.03–1.27), *p* = 0.016). In the adjusted model including common covariates (age, gender, lymphoma subtype) as well as time period (Table 3), risk of death was higher for both U.S. uninsured (HR 1.83 (1.51–2.23), *p* < 0.0001) and Medicaid patients (HR 2.41 (2.09–2.78), *p* < 0.0001) compared to patients with universal coverage (Canada). Survival was slightly improved in privately insured U.S. patients compared to Canadian patients (HR 0.89 (0.79–1.00) *p* = 0.05). Limiting analyses to only those patients ≥ 18 or ≥ 25 years to account for potential differences in treatments for young adults did not substantially change estimates.

### 3.3. Change in Survival over Time

Unadjusted 60 month survival quantified divergence according to insurance status. This demonstrated a large (+7.8%) and small (+2.5%) improvement in Medicaid and universal patients respectively, essentially no change in privately insured (+0.9%) and worse survival (−2.6%) for uninsured patients (Table 4).

## 4. Discussion

Our data demonstrate a heartening improvement in survival for patients with Hodgkin lymphoma over a short period of time, estimated at a 16–20% reduction in death between time periods after adjusting for baseline covariates within both Canada and the U.S. We know that initial deaths are predominantly attributed to relapsed disease and drug toxicity, so there is the potential that improved supportive care or reduced toxicity also contributed to some of this change.

When comparing survival between countries, the U.S. demonstrated an overall higher risk of death compared to Canada. As the majority of U.S. patients were categorized within the private insurance group (approximately 76%) which did not demonstrate a difference in survival compared to universally insured patients, this difference falls to the 25% of patients receiving Medicaid or who were uninsured with a 1.8–2.4 times increased risk of death compared to the referent group. While we can only speculate, this may be due to the inability to afford both expensive medications such as BV and rigorous supportive care. At higher resolution, there was no change in the survival of privately insured U.S. patients, a large improvement in patients on Medicaid, and concernedly worse survival of uninsured patients. Improvements for patients receiving Medicaid may be due to other changes such as improvement in HIV care, which is not captured in the SEER database, or to better coverage for medications like BV [18]. The worse survival for uninsured patients in Period 2 may result from changes in the composition of who was uninsured after the Affordable Care Act was passed in 2010, potentially representing even further marginalized individuals who could not navigate the updated system. However, the percentage of subjects within this group only decreased slightly from 7.8 to 7.2%. Thus, a deeper review of care received and barriers within this group is paramount, though likely to be the most challenging specifically due to the lack of consistent care.

Canadian patients did not see a smaller change in survival improvement compared to privately insured U.S. patients, which suggests that although this delay occurred, either the effect was small enough that it was not captured in this analysis, or other improvements in care moderated any differences seen due to BV access. Potential avenues for receiving therapies prior to funding include compassionate (unfunded) access to BV or limited clinical trial options, which may have been explored more rigorously in Canadian patients due to access delays. However, obtaining compassionate access to unfunded therapies is a complex process requiring negotiation with pharmaceutical companies and infusion centers, often organized by drug access facilitators (employed by larger centers). Therefore, concern for disparity remains in the current Canadian system [10]. Additionally, other therapies were later FDA-approved in the relapsed/refractory setting including Nivolumab in May 2016 and Pembrolizumab in March 2017, though the timing of this relative to when most relapses in HL occur suggests that there would be limited effect on our results.

There are numerous strengths to this dataset. Both SEER and the CCR use similar diagnostic coding and variables allowing for accurate comparison and combination of the two datasets. Given how infrequent events are within this cancer type, datasets were large enough to assess for survival differences, even limited to a short number of years within each time period. SEER is specifically targeted to cover a representative portion of the US population, and CCR covers > 95% of the Canadian population; both undergo rigorous auditing, including vital status, with regular updates. Additionally, while covariates such as comorbidities and chemotherapy are not captured, patients with HL are generally younger with limited comorbidities, and frontline therapy is standardized, with most patients during that period likely receiving ABVD and—less commonly—escBEACOPP.

Limitations to this analysis mainly relate to variables not captured in the databases. Both have limited information on other baseline factors such as comorbidities and accepted variables to classify as favorable or unfavorable risk in limited-stage HL and to compute the international prognostic score (IPS), used in advanced-stage HL. As discussed above, staging data was frequently absent for Canadian patients, limiting sensitivity analysis. Chemotherapy, radiation usage and response assessment on interim or end of treatment PET/CT and relapse timing and management are not recorded in either database for hematologic malignancies, though there are no nationwide databases that capture relapse therapies for cancer. With regards to insurance status, Medicaid may have variable coverage by state, and there is a lack of Medicare or private insurance specificity in SEER dataset; insurance status may also change over time and not be captured after database enrollment. It is also appreciated that individual insurance companies or payers may have had differing times to negotiate fees with the manufacturer of BV. SEER recoded insurance in 2007, precluding inclusion of earlier years of diagnosis. Additionally, the low overall percentage (7.5%) of uninsured patients may limit findings. Lastly, while vital status is routinely updated, cause-specific survival cannot be evaluated from available variables and would be critical to understanding changes in survival over time (i.e., improvement due to new therapies vs. decreased toxicity).

## 5. Conclusions

Overall survival for patients with Hodgkin lymphoma improved by approximately 16–20% in the latter period in the U.S. and Canada. However, significant differences were seen according to insurance status. Uninsured patients had worse survival in Period 2 compared to Period 1, which is concerning and likely not fully related to BV access. Medicaid patients demonstrated poorer survival compared to their peers, but this did improve over time, which may be due to improved treatment of underlying comorbidities such as HIV or hypertension or due to access to therapy. Privately insured patients had stable survival despite presumed access to BV, whereas Canadian (universal) patients had slightly improved survival between periods. However, these differences were not significantly different than privately insured patients in the U.S., and thus, the delay in approval and funding of BV was not felt to have a major impact on the overall trajectory of this disease. The small number of relapsing patients may limit conclusions. However, especially considering the limitations of these cohorts, robust datasets capturing comorbidities, treatment modalities and response, and cause-specific survival are needed.

## Figures and Tables

**Table 1:** Baseline characteristics of patients aged 16–64 years diagnosed with classical Hodgkin lymphoma during Time Periods 1 (2007–2010) and 2 (2011–2014).

	U.S. Time Period 1, *n* = 6204	U.S. Time Period 2, *n* = 5799	Canada Time Period 1, *n* = 2370 *	Canada Time Period 2, *n* = 1840 *
Age, mean years (standard deviation, SD)	35.8 (13.5)	35.7 (13.5)	35.8 (13.7)	34.6 (13.4)
Female (%)	2858 (46.1)	2674 (46.1)	1085 (45.8)	825 (44.8)
Insurance Status (%)			N/A	N/A
Uninsured	482 (7.8)	418 (7.2)		
Any Medicaid	946 (15.3)	1011 (17.4)		
Any Private Insurance	4776 (77.0)	4370 (75.4)		
Race (%)			N/A	N/A
White	5013 (80.8)	4625 (79.8)		
Black	794 (12.8)	752 (13.0)		
Other	351 (5.7)	355 (6.1)		
Missing	46 (0.7)	67 (1.2)		
Spanish–Hispanic–Latino (%)	1029 (16.6)	1012 (17.5)	N/A	N/A
Marital Status (%)			N/A	N/A
Single	2970 (47.9)	2886 (49.8)		
Married, common law, partner	2569 (41.4)	2252 (38.8)		
Separated, divorced, widowed	421 (6.8)	391 (6.7)		
Missing	244 (3.9)	270 (4.7)		
Ann Arbor Stage (%)				
I	872 (14.1)	722 (12.5)	65 (2.7)	45 (2.4)
II	2795 (45.1)	2587 (44.6)	260 (10.9)	260 (14.1)
III	1228 (19.8)	1186 (20.5)	110 (4.6)	100 (5.4)
IV	1103 (17.8)	1150 (19.8)	95 (4.0)	100 (5.4)
Missing	206 (3.3)	154 (2.7)	1845 (77.7)	1330 (72.3)
Lymphoma Subtype (%)				
Nodular sclerosis	4054 (65.3)	3486 (60.1)	1490 (62.9)	1205 (65.4)
Lymphocyte Rich	201 (3.2)	165 (2.9)	95 (4.0)	50 (2.7)
Mixed cellularity	670 (10.8)	595 (10.3)	280 (11.8)	195 (10.6)
Lymphocyte deplete	55 (0.9)	44 (0.8)	.	.
Not otherwise specified	1224 (19.7)	1509 (26.0)	505 (21.3) ^§^	390 (21.2) ^§^
Follow-up time, months (SD)	83.7 (28.8)	42.7 (17.4)	99.2 (29.1)	57.3 (17.4)

*: Canadian data rounded per Statistics Canada requirements. ^§^: Statistics Canada required combination of lymphoma subtype categories “lymphocyte deplete” and “not otherwise specified” for release. N/A = Not available.

**Table 2:** Hazard ratios for death comparing Time Period 2 (2011–2014) vs. 1 (2007–2010) for all US (N = 12,003) and Canadian (N = 4210) patients, stratified by insurance status.

	Unadjusted HR (95% CI)	Adjusted HR (95% CI) *	Adjusted HR (95% CI) **
U.S. Period 2 vs. 1	0.90 (0.80–1.02)	0.87 (0.77–0.98) ^§^	0.80 (0.71–0.91) ^§^
Canadian Period 2 vs. 1	0.79 (0.64–0.97) ^§^	0.84 (0.69–1.03)	
U.S. Patients stratified by insurance status, for period 2 vs. 1			
Uninsured	1.35 (0.94–1.94)	1.21 (0.84–1.75)	1.30 (0.88–1.91)
Any Medicaid	0.59 (0.46–0.74) ^§^	0.53 (0.42–0.67) ^§^	0.50 (0.39–0.64) ^§^
Any Private Insurance	0.99 (0.84–1.15)	0.96 (0.83–1.13)	0.93 (0.79–1.10)

* Adjusted for age, gender, and lymphoma subtype. ** Adjusted for age, gender, stage, race, ethnicity, marital status, and lymphoma subtype. In unstratified analysis, insurance status is also included. ^§^ *p* < 0.05.

**Table 3:** Overall survival by insurance type combining patients from both time periods.

Type of Insurance	HR (95% CI) *	*p* Value
Universal (reference) **		
Uninsured	1.83 (1.51–2.23)	<0.001
Any Medicaid	2.41 (2.09–2.78)	<0.001
Any Private Insurance	0.89 (0.79–1.00)	0.05

* Data adjusted for time period, age, gender, and lymphoma subtype. ** Referring to all Canadian patients.

**Table 4:** Comparison of 60 month unadjusted survival by insurance status, including Canadian and U.S patients for Time Periods 1 and 2.

Type of Insurance	Period 1 (%, 95% CI)	Period 2 (%, 95% CI)	Change in % Survival
Universal *	90.3 (89.1–91.5)	92.8 (91.5–94.1)	+2.5
Uninsured	88.3 (85.3–91.4)	85.7 (81.0–90.4)	+0.9
Any Medicaid	79.3 (76.6–81.9)	87.1 (84.1–90.1)	+7.8
Any Private Insurance	92.1 (91.3–92.8)	93.0 (92.1–93.9)	+0.9

* Referring to all Canadian patients.

## Data Availability

The datasets presented in this article are not readily available because they require a data usage agreement and security clearance (CCR). Requests to access the datasets should be directed to SEER and the CCR.

## Supplementary Materials

The following supporting information can be downloaded at: https://www.mdpi.com/article/10.3390/curroncol31070287/s1, Figure S1: Overall survival of advanced-stage (stage 3/4) U.S. patients with Hodgkin lymphoma diagnosed during Time Period 1 (2007–2010) and Time Period 2 (2011–2014) according to insurance status. Canadian patients were not included as 75.4% lacked staging information.
