# Supplementary material for: Survival Outcomes for US and Canadian Patients Diagnosed with Hodgkin Lymphoma before and after Brentuximab Vedotin Approval for Relapsed/Refractory Disease: A Retrospective Cohort Study
Source: Curr Oncol. 2024 Jul 4;31(7):3885–94. doi: 10.3390/curroncol31070287 (PMC11276208; doi:10.3390/curroncol31070287)
Supplement: Supplementary file 1 [file curroncol-31-00287-s001.zip › curroncol-3057615-supplementary.pdf]

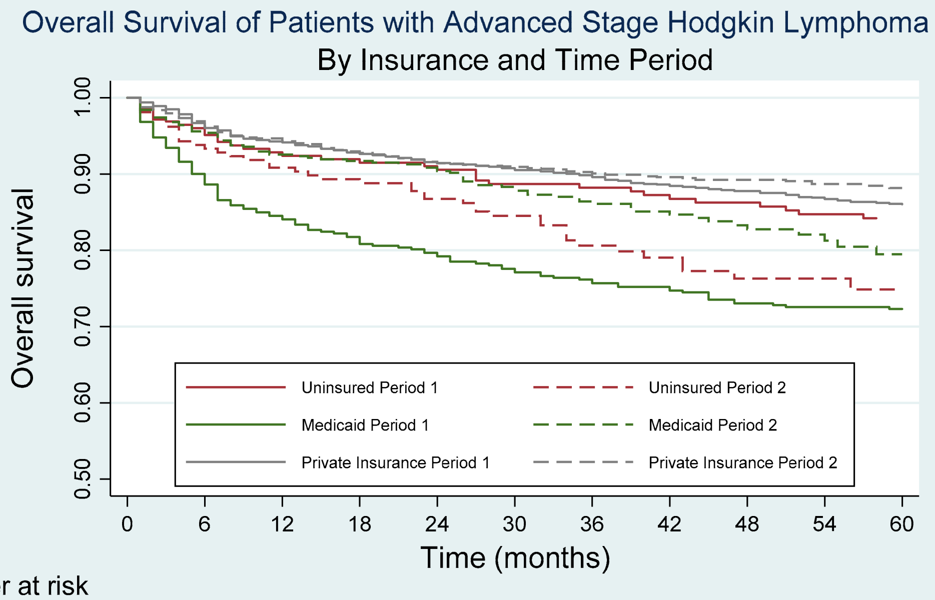

Number at risk

Uninsured Period 1	226	214	204	200	198	187	184	178	173	166	163
Uninsured Period 2	211	192	181	174	167	143	113	92	74	60	38
Medicaid Period 1	442	394	368	354	343	331	321	313	302	298	289
Medicaid Period 2	504	474	445	431	411	351	279	215	155	105	68
Private Insurance Period 1	1663	1604	1561	1536	1512	1490	1469	1442	1424	1403	1372
Private Insurance Period 2	1621	1563	1506	1454	1408	1203	997	823	625	447	281

Figure S1. Overall survival of advanced-stage (stage 3/4) U.S. patients with Hodgkin lymphoma diagnosed during Time Period 1 (2007–2010) and Time Period 2 (2011–2014), according to insurance status. Canadian patients not included as 75.4% lacked staging information.
